# Incidence, Costs and Predictors of Non-Union, Delayed Union and Mal-Union Following Long Bone Fracture

**DOI:** 10.3390/ijerph15122845

**Published:** 2018-12-13

**Authors:** Christina L. Ekegren, Elton R. Edwards, Richard de Steiger, Belinda J. Gabbe

**Affiliations:** 1 Department of Epidemiology and Preventive Medicine, Monash University, Melbourne VIC 3004, Australia; ere@bigpond.net.au (E.R.E); belinda.gabbe@monash.edu (B.J.G.); 2 Department of Orthopaedic Surgery, Alfred Hospital, Melbourne VIC 3004, Australia; 3 Department of Surgery, Epworth Healthcare, Richmond VIC 3121, Australia; richard.desteiger@epworth.org.au; 4 Department of Surgery, University of Melbourne, Parkville VIC 3052, Australia; 5 Health Data Research UK, Swansea University, Swansea SA2 8PP, UK

**Keywords:** bone, fracture, non-union, delayed union, mal-union, data linkage, costs

## Abstract

Fracture healing complications are common and result in significant healthcare burden. The aim of this study was to determine the rate, costs and predictors of two-year readmission for surgical management of healing complications (delayed, mal, non-union) following fracture of the humerus, tibia or femur. Humeral, tibial and femoral (excluding proximal) fractures registered by the Victorian Orthopaedic Trauma Outcomes Registry over five years (*n* = 3962) were linked with population-level hospital admissions data to identify two-year readmissions for delayed, mal or non-union. Study outcomes included hospital length-of-stay (LOS) and inpatient costs. Multivariable logistic regression was used to determine demographic and injury-related factors associated with admission for fracture healing complications. Of the 3886 patients linked, 8.1% were readmitted for healing complications within two years post-fracture, with non-union the most common complication and higher rates for femoral and tibial shaft fractures. Admissions for fracture healing complications incurred total costs of $4.9 million AUD, with a median LOS of two days. After adjusting for confounders, patients had higher odds of developing complications if they were older, receiving compensation or had tibial or femoral shaft fractures. Patients who are older, with tibial and femoral shaft fractures should be targeted for future research aimed at preventing complications.

## 1. Introduction

Fractures are the most common form of hospitalised trauma, contributing to over 150,000 hospitalisations and almost one million bed-days in Australia each year [1]. In the United States (US), direct costs for healthcare and loss of productivity in the first six months post-injury are estimated at up to $23,000 per isolated limb fracture [2]. Fractures of the femoral shaft, humerus and tibia account for 3%, 14% and 24% of fractures in working-age adults, respectively, and are commonly the result of hi-energy trauma such as a fall from height and transport accidents [3]. The management of these long bone fractures is complex, and the risk of mal-union, delayed union and non-union remains high, contributing to considerable patient disability, reduced quality of life and significant treatment costs [4,5].

Rates of non-union of 5–10% have been reported but many estimates are from small, single-site studies using a variety of definitions, and the overall rate of non-union remains unclear [6,7]. Specific anatomical areas known to have a higher incidence of non-union than others include the humerus, femur and tibia. A recent population-based study from Scotland estimated the incidence of non-union at 13 per 1000 fractures per annum for the pelvis and femur, 30 per 1000 fractures per annum for the humerus and approximately 55 per 1000 fractures per annum for the tibia and fibula [6]. However, by averaging rates across adjacent bones (e.g., pelvis and femur) this study provided uncertain estimates.

In the United Kingdom (UK), the cost of treatment of non-union has been estimated at £7000 to £79,000, but these figures relate only to the cost of hospital treatment [8,9]. In the US, the median cost of treating fracture non-unions has been estimated at $25,556 USD per open tibial fracture, with increased healthcare utilisation, and increased prescription and longer duration of opioids in these cases [10]. Given the differences in the healthcare systems and funding models across countries, direct extrapolation of cost estimates from other healthcare jurisdictions is difficult, and local estimates are needed.

Previous studies have attempted to identify factors associated with fracture healing complications, allowing for better targeting of patient monitoring and management. There are reports of lower rates of non-union amongst older adults, although this may relate to the fact that non-union is more likely following high-energy trauma, which is less common in older adults [6]. Other hypothesised risk factors include male sex, smoking, diabetes and poor operative fixation, although these have not been clearly quantified with several studies marred by under-powering and inadequate control for confounding [11,12].

Considering conflicting rates of fracture healing complications, costs of treatment and predictors of healing complications reported within the literature, more research is needed from large, multi-site studies, in discrete anatomical sites, with clearly defined end-points. A well-accepted definition of non-union is a fracture that in the opinion of the treating physician has no possibility of healing without further intervention [13]. Determining hospital readmissions for surgical management of healing complications can provide close estimates of cases meeting this definition. By linking a multisite orthopaedic trauma registry with population-level hospital readmissions data, we aimed to determine (i) the rate of two-year readmission for fracture healing complications following fracture of the proximal and shaft of humerus, subtrochanteric, shaft and distal femur, and shaft of tibia (ii) time to readmission, costs and length of stay of hospitalisation for fracture complications in the first two years; and (iii) demographic and injury-related factors associated with readmission for fracture healing complications.

## 2. Methods

A retrospective cohort study of cases was undertaken using data from the Victorian Orthopaedic Trauma Outcomes Registry (VOTOR). The VOTOR is a sentinel site clinical registry capturing data about all adult (≥16 years) orthopaedic trauma admissions to four hospitals in Victoria [14]. Any emergency admission to these hospitals, with an orthopaedic injury in the International Classification of Disease, 10th revision, Australian Modification (ICD-10-AM) [15] principal diagnosis code list, and a hospital length of stay >24 h is included on the registry. Patients are excluded from the registry if they have a fracture related to metastatic disease. The registry has been collecting data since 2003 and now captures approximately 7000 cases per year, with an opt-out rate of less than 2% [14]. For this study, all patients with a proximal or shaft of humerus, proximal, distal or shaft of femur, or shaft of tibia fracture registered by VOTOR (Supplementary Material 1: ICD-10-AM codes) with a date of admission from 1 January 2007 to 31 December 2011 were included. Ethics approval for this study was obtained from each participating hospital from December 2014 to February 2015 (The Alfred: 575/14; Barwon Health: 14/164; Royal Melbourne Hospital: QA2014213l; Northern Health: LR01.2015).

The VOTOR dataset was submitted to the Victorian Department of Health and Human Services for linkage with the Victorian Admitted Episodes Dataset (VAED). The VAED comprises data on all admissions to all Victorian public and private hospitals including acute care, rehabilitation, extended care, and day procedure centres. The VAED and VOTOR data were linked using probabilistic linkage (via hospital and hospital UR number, patient name, date of birth, gender, admission date and discharge date). Admissions from 1 January 2007 until 31 December 2013 (allowing for readmissions up to two years post-fracture) were included in the linked dataset.

Following receipt of linked data, case selection was undertaken to ensure only relevant readmissions were included. We excluded: (i) duplicate admissions, (ii) admissions that represented direct transfers to rehabilitation, (iii) hospital admissions that were before or more than two years after the index fracture admission, and (iv) admissions without an ICD-10-AM diagnosis of non-union, mal-union or delayed union (Supplementary Material 1). We also excluded admissions for different fractures to the original index fracture by inspecting surgical procedure codes. Where a general, rather than body area-specific, surgical code was recorded, we used Australian National Diagnosis Related Groups (AN-DRG) surgical codes to verify the body region operated on [16].

Population descriptors extracted from the linked dataset included: demographic information (sex, age, marital status); postcode of residence mapped to the Accessibility/Remoteness Index of Australia (ARIA) (a geographical index of remoteness), and the Index of Relative Socio-economic Advantage and Disadvantage (IRSAD) (an index of relative socio-economic advantage and disadvantage); pre-injury level of disability (self-reported using World Health Organization definition) [17]; presence of comorbidities (defined using the Charlson Comorbidity Index (CCI), mapped from ICD-10-AM comorbid conditions, with a CCI of zero representing no CCI conditions) [18]; injury cause and diagnosis (ICD-10-AM codes); and compensable status. Compensable status was classified as (i) Non-compensable/Medicare, (ii) Private health insurance or Department of Veteran’s Affairs (DVA); or (iii) Compensable (WorkSafe Victoria or Transport Accident Commission (TAC)). Medicare is Australia’s public healthcare system which provides universal coverage for all Australian citizens and permanent residents; private health insurance is held by approximately 57% of Australian adults [19], and 46% of injured patients [20]; the DVA provides financial support for war veterans and their dependants, members of the Australian Federal Police and Australian Defence Force personnel; and WorkSafe Victoria and the TAC and are the no-fault, third party insurers for work and transport injury, providing compensation for treatment, income replacement and long-term care services.

Outcomes of interest included: (i) proportion of cases with each complication of interest defined as the number of cases readmitted to hospital with non-union, mal-union or delayed union divided by the number of cases overall; (ii) months to readmission; (iii) hospital length of stay for treatment of healing complication, defined as the total number of bed days in an acute hospital; and (iv) inpatient costs of treating the fracture healing complication (in AUD for relevant year), derived using a case-mix approach based on AN-DRGs [16]. The AN-DRG classifies inpatient admissions into clinically meaningful categories of similar levels of complexity that consume similar amounts of resources (e.g., removal of internal fixation in the hip/femur, knee replacement, etc.). These classifications were provided by the Victorian Department of Health and Human Services for each linked hospital admission, and cross-referenced with National Hospital Cost Data Collection Cost Weight Tables [21] for each relevant year.

Demographic characteristics were categorised for analysis (age group, sex, marital status, ARIA, IRSAD decile, comorbidities (CCI), cause of injury, compensable status, head injury, other non-orthopaedic injuries and fracture type) and compared between patients with and without fracture complications using logistic regression. Variables showing a significant (*p* < 0.25) difference between groups on preliminary univariable analyses, in addition to those deemed clinically important (age group and gender), were entered into the multivariable logistic model [22]. Adjusted odds ratios (AORs) with 95% confidence intervals are reported for patients readmitted for healing complications. All analyses were performed using Stata Version 15 (StataCorp, College Station, TX, U.S.) and an a priori alpha level of 0.05 used for all tests.

## 3. Results

### 3.1. Patients Readmitted for Fracture Healing Complications

**Figure 1:**
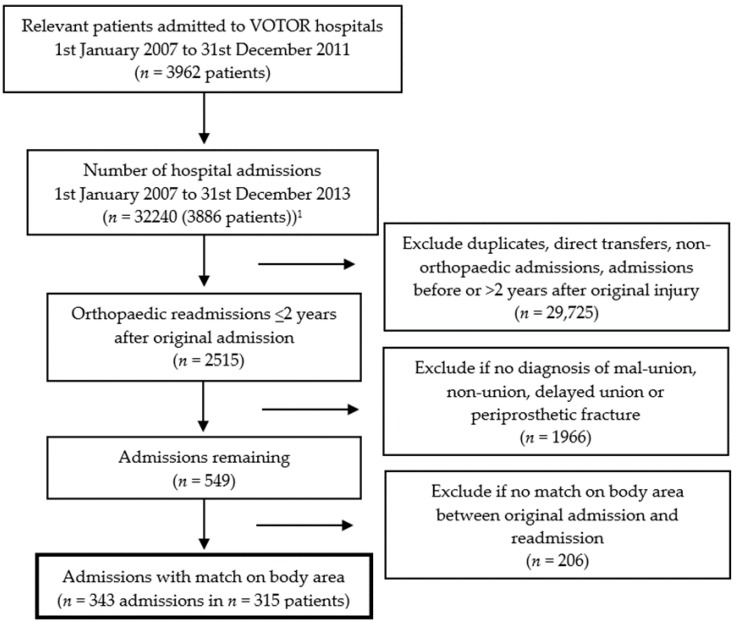
Case selection flow chart. ^1^ 98% linkage.

From 1 January 2007 to 31 December 2011, there were 3962 relevant fracture patients registered by VOTOR, 98% of which (*n* = 3886) were able to be linked with patients admitted to public and private hospitals within Victoria from 1 January 2007 to 31 December 2013 (Figure 1). Out of the original 3886 linked patients, 8.1% (*n* = 315) were readmitted to hospital for fracture healing complications (i.e., non-union, mal-union and delayed union) within two years of their index fracture admission. The most common type of complication resulting in readmission was non-union (*n* = 264, 6.8%). Other types of fracture healing complications included mal-union (29, 0.7%) and delayed union *n* = 22, 0.6%). All readmissions involved orthopaedic surgical care.

### 3.2. Time to Hospital Readmission, Length of Stay and Costs for Fracture Healing Complication Admissions

The median (IQR) time to readmission following the original fracture was 8 (5–12) months (Table 1). The median (IQR) hospital length of stay for all complication admissions was 2 (1–5) days. The total inpatient costs for all admissions for fracture healing complications in this cohort (*n* = 315) was $4,916,438 AUD. The median inpatient hospital cost per patient for all complication admissions was $14,957 AUD.

### 3.3. Type and Number of Readmissions for Fracure Healing Complications

The majority (92%) of patients experiencing fracture healing complications were admitted once within two years of their index fracture (Table 2). However, 8% had two admissions for healing complications during this period, and one patient had three admissions. Out of all fractures, proximal humerus fractures and shaft of tibia fractures were most the common types of fracture. The highest rate of complication readmissions was in patients with femoral shaft fractures (13.6%), followed by tibial shaft fractures (11.7%). The lowest complication readmission rate was in proximal humerus fractures (2.3%).

### 3.4. Characteristics of Patients Admitted for Fracture Healing Complications

Patients admitted for complications were younger and more commonly male (Table 3). Patients with complications were more commonly from outer regional and remote parts of Australia, injured via road trauma and compensated by a third-party insurer for work or transport accidents when compared to patients without complications. It was also more common for patients with complications to have head injuries and other non-orthopaedic injuries, such as abdominal and chest injuries, compared to those without complications.

### 3.5. Factors Associated with the Admission for Fracture Healing Complications

As noted, there were differences between the groups who were and were not admitted to hospital for management of fracture healing complications in the first two years following injury. In the adjusted multivariable model, age group, compensable status and fracture type were significant predictors of admission for fracture healing complications within two years of injury (Table 4). Specifically, the adjusted odds of admission for fracture healing complications were more than two-fold higher for patients aged 55–64 years compared to those aged 15–24 years. Similarly, patients receiving compensation demonstrated more than double the adjusted odds of readmission for fracture healing complications when compared to non-compensable patients. Compared to fractures of the proximal humerus, all other fracture types demonstrated adjusted odds of fracture healing complications resulting in hospital readmission between two- and almost five-fold higher, with the greatest odds of readmission for shaft of femur fractures (AOR (95%CI): 4.67 (2.91,7.50)).

## 4. Discussion

The key purpose of this study was to describe the incidence, inpatient costs, length of stay and predictors of hospitalisation for surgical management of healing complications following fracture of the humerus, tibia or femur. Of the 3886 patients included in the study, 8% were admitted to hospital for fracture healing complications within two years of their index fracture. The total cost of hospitalisations for these complications was AUD $4.9 M. Factors that predicted the development of complications requiring readmission, included being older, receiving compensation and having any fracture type other than a proximal humerus fracture, with shaft of femur and shaft of tibia fractures having the greatest odds of readmission.

Comparing rates of fracture healing complications between studies is challenging because the majority of previous research has focused on small cohorts with specific fracture types, specific surgical repair methods, and varying outcome definitions. While rates of non-union of 5–10% are commonly reported [8], and are consistent with our results, a comprehensive review of studies of non-union in long bones reported non-union rates of 0–12% in femoral fractures, 0–33% in humeral fractures and 1–80% in tibial fractures [23]. A population-based study conducted in Scotland reported non-union rates of only 1.9% per annum, with the highest incidence of non-union in the lower leg for the majority of age groups (5.5% per annum) [6]. In line with our data, a large study (*n* = 853 patients), reported non-union rates in tibial shaft fractures of 12% over a two-year period [10].

Cost estimations for fracture healing complications also vary widely in the existing literature, depending on the type of complication studied and the method of cost analysis. A review of evidence on treatment costs for long-bone fracture non-unions conducted in the UK, reported total costs of £15,566 ($27,100 AUD), £17,200 ($29,944 AUD) and £16,330 GBP ($28,429 AUD) per humeral, femoral, and tibial non-union, respectively [9]. In the US, treating a non-united open tibial fracture was estimated at $25,556 USD ($34,472 AUD) per patient, inclusive of inpatient, outpatient and pharmaceutical costs [10]. When converted to Australian dollars, these values were higher than values reported in our study. However, we only included inpatient costs of admissions, and not out-of-hospital costs.

Similar to previous research, we found that older age was associated with an increased odds of admission for healing complications, potentially due to reduced bone density in this population [24]. Conversely, Mills et al. [6] reported a higher rate of non-union in middle-aged than older adults. However, they did not adjust for the potential confounding effect of injury mechanism; high-trauma fractures have a higher risk of non-union and are also more prevalent in middle-aged than older adults [11,12]. Previously, male sex has been associated with an increased risk of non-union [25]. While there were higher rates of non-union in our male participants, this association was not apparent once we controlled for confounding. The same was true for comorbidity, which has been associated with non-union in previous studies [10,25]. Our reliance on the CCI, which does not apply a weighting for osteoporosis, or reduced bone mineral density, may have led to an inability to adjust for all comorbidities of relevance. However, the CCI does include diabetes, which has also previously been associated with non-union risk [25]. A novel finding was the increased risk of healing complications amongst patients receiving compensation for their injuries, either from road trauma or workplace schemes. Receiving compensation has previously been linked to poorer outcomes following injury possibly due to reduced financial imperative to recover or greater injury severity [26,27,28]. It might also be the case that the availability of funding for treatment results in more rapid decision making on the need for further surgery or shorter surgical waiting times. However, it is also possible that this association was confounded by injury severity, a factor for which data limitations precluded adjustment.

A potential study limitation was that our case definition focused solely on patients admitted for surgical management of healing complications within two years of their initial fracture. Other, generally small-scale studies, have used radiological and/or clinical methods for defining mal-union, non-union and delayed union, e.g., persistent pain and functional impairment [12,29]. However, there is still a lack of consensus in the literature about how to define fracture healing complications and wide variability in the inter- and intra-rater reliability of these methods [29,30]. Therefore, although we may have missed cases of non-union, mal-union or delayed union which were managed conservatively or not diagnosed within the two-year period, using a definition based on surgical management allows for reliable capture of those cases deemed severe enough to warrant further surgery. This study focused on humeral, femoral (excluding femoral neck), and tibial fractures. However, fracture healing complications have been shown to be a problem with other fracture types such as ulnar, radial and clavicular fractures, which we did not include [6]. Finally, the observational nature of our study precludes attribution of causation and, owing to data limitations, there were potential risk factors for healing complications that we were not able to include in our predictive model, such as fracture type (i.e., open/closed), fracture history, smoking, drugs, other lifestyle factors, serum levels of calcium, Vitamin D status, and osteoporosis.

## 5. Conclusions

Our study has provided important new knowledge of fracture healing complication rates in a large, multi-site sample. We have provided local estimates of costs and length of stay attributed to treating patients with non-union, delayed union and mal-union. The findings of this study have provided the data necessary to drive prioritisation of future research towards preventing fracture healing complications, particularly in patients who are older, receiving compensation and with fractures of the tibial and femoral shafts.

## Figures and Tables

**Table 1:** Time to hospital readmission, length of stay and costs for fracture healing complication admissions within two years of original fracture (*n* = 343 admissions for *n* = 315 patients).

		1st Admission	All Admissions (Range 1–3 per Patient)
Time to Admission (months)	Range per patient	2–24	
	Median (IQR) per patient	8 (5–12)	
Length of stay (days)	Range per patient	1–63	1–109
	Median (IQR) per patient	2 (1–5)	3 (1–6)
	Total days all patients	1549	1792
Costs ($AUD)	Range per patient	$4788–$35,890	$4788–$93,197
	Median (IQR) per patient	$14,957 ($9462–$15,520)	$14,957 ($9632–$19,887)
	Total costs all patients	$4,339,098	$4,916,438

**Table 2:** Fracture healing complication admissions within two years of index fracture—by fracture type (*n* = 3886 patients).

Fracture Type	Fractures (*n*)	Admissions per Patient (*n*)			Total Admissions (*n*, % Fractures)
		1	2	3	
Proximal humerus fracture	1117	26			26 (2.3)
Shaft of humerus fracture	413	28	2		32 (7.8)
Subtrochanteric fracture	308	13			13 (4.2)
Shaft of femur fracture	791	89	9		107 (13.5)
Distal femur fracture	465	25	7		39 (8.4)
Shaft of tibia fracture	1075	111	6	1	126 (11.7)
Total	4169 ^1^				343 (8.2)

^1^ Total fractures greater than total patients due to multiple fractures in 209 patients.

**Table 3:** Characteristics of patients with and without fracture healing complications (*n* = 3,886).

Descriptor		Patients with Complications (*n* = 315) *n* (%)	Patients without complications (*n* = 3571) *n* (%)
Age group	15–24 years	61 (19.4)	566 (15.9)
	25–34 years	61 (19.4)	475 (13.3)
	35–44 years	49 (15.6)	418 (11.7)
	45–54 years	41 (13.0)	364 (10.2)
	55–64 years	53 (16.8)	396 (11.1)
	65–74 years	23 (7.3)	465 (13.0)
	75–84 years	20 (6.4)	551 (15.4)
	85+ years	7 (2.2)	336 (9.4)
Sex	Male	221 (70.2)	1936 (54.2)
	Female	94 (29.8)	1635 (45.8)
Marital status	Never married	116 (36.8)	1183 (33.1)
	Widowed	19 (6.0)	536 (15.0)
	Divorced/Separated	14 (4.4)	225 (6.3)
	Married	122 (38.7)	1281 (35.9)
	De Facto	24 (7.6)	203 (5.7)
	Not stated/inadequately described	20 (6.4)	143 (4.0)
ARIA ^1^	Major cities of Australia	221 (70.8)	2592 (74.4)
	Inner regional Australia	70 (22.4)	769 (22.1)
	Outer regional and remote Australia	21 (6.7)	121 (3.5)
IRSAD decile ^2^	10 (Most advantaged)	40 (12.8)	582 (16.7)
	9	4450 (16.0)	570 (16.4
	8	46 (11.5)	570 (16.4)
	7	33 (14.1)	462 (13.3)
	6	33 (10.6)	274 (7.9)
	5	28 (9.0)	238 (6.8)
	4	25 (8.0)	266 (7.6)
	3	21 (6.7)	100 (2.9)
	2	19 (6.1)	219 (6.3)
	1 (Most disadvantaged)	16 (5.1)	203 (5.8)
Comorbidities (CCI)	None	235 (74.6)	2545 (71.3)
	CCI = 1	67 (21.3)	736 (20.6)
	CCI > 1	13 (4.1)	290 (8.1)
Cause of injury	Low fall	47 (14.9)	1350 (37.8)
	High fall	23 (7.3)	296 (8.3)
	Road trauma	209 (66.4)	1,401 (39.2)
	Other external cause	36 (11.4)	524 (14.7)
Compensable status ^3^	Medicare/Non compensable	74 (23.6)	1737 (48.9)
	Private/DVA	30 (9.6)	515 (14.5)
	TAC/Worksafe/Other compensable	210 (66.9)	1301 (36.6)
Head injury	No	240 (76.2)	3036 (85.0)
	Yes	75 (23.8)	535 (15.0)
Other non-orthopaedic injuries	No	194 (61.6)	2729 (76.4)
	Yes	121 (38.4)	842 (23.6)
Fracture readmission type	Proximal humerus	26 (8.3)	1021 (28.6)
	Shaft of humerus	30 (9.5)	320 (9.0)
	Subtrochanteric	13 (4.1)	263 (7.4)
	Shaft of femur	97 (30.8)	560 (15.7)
	Distal femur	31 (9.8)	352 (9.9)
	Shaft of tibia	116 (36.8)	848 (23.8)
	Shaft of tibia and femur	2 (0.6)	207 (5.8) ^4^

^1^ Data missing for 92 patients; ^2^ Data missing for 90 patients; ^3^ Data missing for 19 patients; ^4^ Value represents all patients with fractures in multiple body areas; ARIA, Accessibility/Remoteness Index of Australia; IRSAD, Index of Relative Socio-economic Advantage and Disadvantage; CCI, Charlson Comorbidity Index; DVA, Department of Veterans Affairs; TAC, Transport Accident Commission.

**Table 4:** Factors associated with the admission for fracture healing complications (multivariable analyses).

Descriptor		Multivariable Analysis (*n* = 3775)	
		AOR (95% CI)	P
Age group	15–24 years	1.00 (ref.)	0.004
	25–34 years	1.37 (0.91,2.07)	
	35–44 years	1.28 (0.81,2.02)	
	45–54 years	1.49 (0.91,2.46)	
	55–64 years	2.44 (1.48,4.02)	
	65–74 years	1.20 (0.64,2.22)	
	75–84 years	0.99 (0.50,1.97)	
	85+ years	0.67 (0.25,1.80)	
Compensable status ^1^	Medicare/Non compensable	1.00 (ref.)	<0.001
	Private/DVA	1.41 (0.90,2.22)	
	TAC/Worksafe/Other compensable	2.43 (1.54,3.83)	
Fracture type	Proximal humerus readmission	1.00 (ref.)	<0.001
	Shaft of humerus readmission	2.81 (1.60,4.92)	
	Subtrochanteric readmission	2.62 (1.31,5.26)	
	Shaft of femur readmission	4.67 (2.91,7.50)	
	Distal femur readmission	2.56 (1.47,4.45)	
	Shaft of tibia readmission	3.39 (2.13,5.38)	
	Shaft of tibia and femur readmission	0.20 (0.05,0.88)	

^1^ Data missing for 19 patients; AOR, adjusted odds ratio; DVA, Department of Veterans Affairs; TAC, Transport Accident Commission.

## Supplementary Materials

The following are available online at http://www.mdpi.com/1660-4601/15/12/2845/s1, Table S1: ICD-10-AM codes for included fractures and diagnoses of mal, non and delayed union.
