# Supplementary material for: Incidence, Costs and Predictors of Non-Union, Delayed Union and Mal-Union Following Long Bone Fracture
Source: Int J Environ Res Public Health. 2018 Dec 13;15(12):2845. doi: 10.3390/ijerph15122845 (PMC6313538; doi:10.3390/ijerph15122845)
Supplement: Supplementary file 1 [file ijerph-15-02845-s001.pdf]

International Journal of
Environmental Research and Public Health

MDPI

Supplementary Material 1. ICD-10-AM codes for included fractures and diagnoses of mal, non and delayed union

Diagnosis	ICD-10-AM code
**Fracture of the proximal humerus**	**S42.2**
Fracture of the upper end of the humerus (part unspecified)	S42.20
Fracture of the humeral head	S42.21
Fracture of the surgical neck	S42.22
Fracture of the anatomical neck	S42.23
Fracture of the greater tuberosity	S42.24
Fracture of other and multiple parts of upper end of humerus	S42.29
**Fracture of the shaft of humerus**	**S42.3**
**Subtrochanteric fracture of the femur**	**S72.2**
**Fracture of the shaft of the femur**	**S72.3**
**Fracture of the lower end of the femur**	**S72.4**
Fracture of lower end of femur (part unspecified)	S72.40
Femoral condyle	S72.41
Supracondylar fracture	S72.43
Intercondylar fracture	S72.44
**Fracture of shaft of tibia**	**S82.2**
Fracture of shaft of tibia with fracture of fibula	S82.21
Other fracture of shaft of tibia	S82.28
**Mal-union of fracture**	**M84.0**
Mal-union of fracture (multiple sites)	M84.00
Mal-union of fracture (shoulder region)	M84.01
Mal-union of fracture (upper arm)	M84.02
Mal-union of fracture (pelvic region and thigh)	M84.05
Mal-union of fracture (lower leg)	M84.06
Mal-union of fracture (ankle and foot)	M84.07
Mal-union of fracture (site unspecified)	M84.09
**Non-union of fracture**	**M84.1**
Non-union of fracture (multiple sites)	M84.10
Non-union of fracture (shoulder region)	M84.11
Non-union of fracture (upper arm)	M84.12
Non-union of fracture (pelvic region and thigh)	M84.15
Non-union of fracture (lower leg)	M84.16
Non-union of fracture (ankle and foot)	M84.17
Non-union of fracture (site unspecified)	M84.19
**Delayed union of fracture**	**M84.2**
Delayed union of fracture (multiple sites)	M84.20
Delayed union of fracture (shoulder region)	M84.21
Delayed union of fracture (upper arm)	M84.22
Delayed union of fracture (pelvic region and thigh)	M84.25
Delayed union of fracture (lower leg)	M84.26
Delayed union of fracture (ankle and foot)	M84.27
Delayed union of fracture (site unspecified)	M84.29
